# Progressive Rehabilitation Based on EMG Gesture Classification and an MPC-Driven Exoskeleton

**DOI:** 10.3390/bioengineering10070770

**Published:** 2023-06-27

**Authors:** Daniel Bonilla, Manuela Bravo, Stephany P. Bonilla, Angela M. Iragorri, Diego Mendez, Ivan F. Mondragon, Catalina Alvarado-Rojas, Julian D. Colorado

**Affiliations:** 1 School of Engineering, Pontificia Universidad Javeriana, Bogota 110231, Colombia; daniel-bonillab@javeriana.edu.co (D.B.); m-bravo@javeriana.edu.co (M.B.); stephany.bonilla@javeriana.edu.co (S.P.B.); diego-mendez@javeriana.edu.co (D.M.); imondragon@javeriana.edu.co (I.F.M.); catalina_alvarado@javeriana.edu.co (C.A.-R.); 2 Neurology, School of Medicine, Hospital Universitario San Ignacio, Bogota 110231, Colombia; airagorri@javeriana.edu.co

**Keywords:** stroke, electromyography (EMG), exoskeleton, rehabilitation, gesture classification, model predictive control (MPC)

## Abstract

Stroke is a leading cause of disability and death worldwide, with a prevalence of 200 millions of cases worldwide. Motor disability is presented in 80% of patients. In this context, physical rehabilitation plays a fundamental role for gradually recovery of mobility. In this work, we designed a robotic hand exoskeleton to support rehabilitation of patients after a stroke episode. The system acquires electromyographic (EMG) signals in the forearm, and automatically estimates the movement intention for five gestures. Subsequently, we developed a predictive adaptive control of the exoskeleton to compensate for three different levels of muscle fatigue during the rehabilitation therapy exercises. The proposed system could be used to assist the rehabilitation therapy of the patients by providing a repetitive, intense, and adaptive assistance.

## 1. Introduction

Stroke is a cerebrovascular disease characterized by an insufficient blood supply to the brain due to a blockage (ischemic stroke) or rupture (hemorraghic stroke) of blood vessels [1]. The consequence of the brain damage could be long-term disability or even death. According to the World Health Organization (WHO), around one in four people is estimated to have a stroke in their lifetime, which is the principal cause of disability worldwide and the second leading cause of death [2]. Motor impairment of the face or upper or lower extremities after stroke is presented in about 80% of patients [3]. The motor dysfunction commonly includes muscle weakness, changes in muscle tone, and impaired motor control [4,5]. In particular, the disability of the hand poses limitations to carry out activities of daily life (ADLs), affecting the independence of the patient [4,6]. Recovery of up to 70% of the motor function is possible with rehabilitation in the first 3 to 6 months after the stroke event [7]. The hand, however, requires longer rehabilitation periods and reaches lower recovery levels [8]. This challenge is partly due to the complex biomechanics of the hand [9]. Maximal functional recovery requires a repetitive, intensive, and task-oriented therapy [10], to repair muscle weakness and spasticity, somatosensory loss, and abnormal coactivation of muscles during isolated finger movements [11,12].

In order to improve the rehabilitation outcome, different neurotechnologies have been proposed in the last decade [13,14,15,16]. The use of robotics, brain–computer interfaces, and noninvasive stimulation has showed promising results and needs to be further explored to translate them to clinical practice [1,16,17]. In particular, prostheses and orthoses, such as robotic exoskeletons, could enhance rehabilitation outcomes by facilitating precise motion control and real-time monitoring of different variables during therapy.

The data-driven control of the exoskeleton can be performed with mechanical or electrical signals. The electrical signals include electromyography (EMG), which measures the electrical muscle activity in response to a movement command from the central nervous system. The muscles involved in the fingers movement are located in the forearm (extrinsic muscles) for coarse movements and in the hand (intrinsic muscles) for fine movements. For instance, the flexion or extension of fingers is a combination of the activation of the extrinsic muscles: flexor digitorum superficialis, flexor digitorum profundus, and extensor digitorum communis [18,19]. The superficial EMG can partially measure the activity of the superficial muscles, and can detect the corresponding movement by means of signal processing and machine learning (ML) techniques [20,21]. A standardized methodology for EMG hand gesture recognition has been recently proposed, including the following steps: data acquisition, data preprocessing, data segmentation, feature extraction, classification, and performance evaluation [21].

Data acquisition consists of placing electrodes on the skin to record the electrical activity of the muscles of interest. Different studies have used EMG systems, such as Delsys Trigno EMG system [22,23,24,25] and Myo Armband [6,26,27,28,29]. The recorded raw data can be filtered to reject noise and segmented in overlapped or nonoverlapped windows, each containing an individual movement [21]. Subsequently, feature extraction in time, frequency, or time–frequency domains is implemented. Time domain features are efficient and can be usually implemented in real time. The most common measures are mean absolute values (MAV), root mean square (RMS), zero-crossing (ZC), waveform length (WL), and Willison amplitude (WA), among others [21,30]. Frequency domain features include measures from the fast Fourier transform or power spectrum of the signal, such as mean frequency, peak frequency, frequency ratio, and total power, among others [21,31,32,33,34]. On the other hand, time–frequency features include short-time Fourier transform and wavelet transform [32,35,36,37]. The classifiers that have presented the best results are artificial neural networks (ANs) [23,38], k nearest neighbors (kNNs), [6,39], linear discriminant analysis (LDA), [32,40], support vector machines (SVM) [41,42], and deep learning models such as convolutional neural networks (CNNs) [24,27,29,43]. The performance of different machine learning and deep learning techniques for the classification of finger gestures from EMG signals have obtained an average accuracy between 87% for deep learning and 94% for machine learning techniques (Sultana 2023). Despite the huge amount of work in gesture recognition with EMG signals, there are very few methods in real-time classification of gestures on embedded systems.

Once the intention of movement has been detected through machine learning techniques, the exoskeleton should guide the movement of the hand in the corresponding therapy. It is then necessary to have a control of the movement of the exoskeleton to facilitate the human–robot interaction. In order to promote brain plasticity and foster recovery, the exoskeleton function should be to assist the desired movement. In addition to the tracking of a specific trajectory, the compensation for fatigue effects that can suffer the patient during a single therapy has been minimally studied [44]. Furthermore, assist-as-needed exoskeleton of the upper limb has been proposed specially for shoulder and wrist, with very few studies on the hand and finger movements [45], among assist-as-needed exoskeletons of the hand [44,46]. In this regard, the model predictive control (MPC) is a popular technique with the ability to handle constraints, optimizing the control output while considering the future states of the exoskeleton [47].

**Figure 1:**
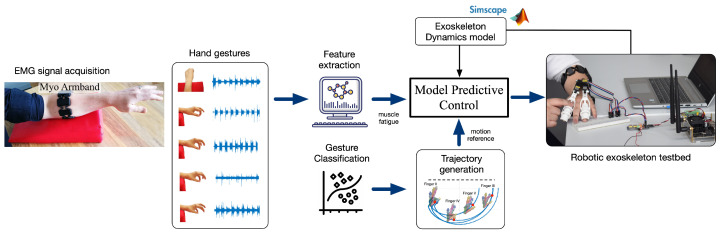
System architecture.

Motivated by the potential use of robotic exoskeleton systems to assist in the recovery of hand motor function, our study proposes a system for real-time identification of hand gestures from EMG signals implemented in an embedded system. In addition, a closed-loop control system was developed in an exoskeleton with the aim of compensating the effects of muscle fatigue in hand joint rehabilitation therapies. A model predictive control technique is implemented to drive the exoskeleton by following a joint trajectory given by the therapy, but also considering an estimation of the driven velocity according to the EMG-based muscular effort detected within the control loop. Figure 1 details the proposed system.

## 2. Materials and Methods

### 2.1. Data Acquisition

**Figure 2:**
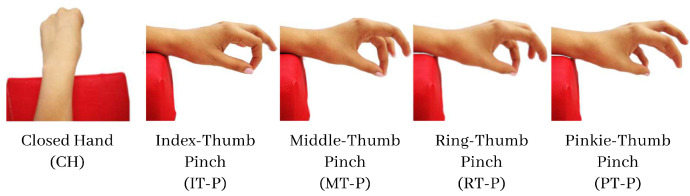
Gestures of interest in rehabilitation therapy. Modified from previous work in [51].

In this study, we selected five movements of interest, due to their importance in hand dexterity and fine motor skills: index-thumb pinch (IT-P), middle-thumb pinch (MT-P), ring-thumb pinch (RT-P), pinkie-thumb pinch (PT-P), and closed hand (CH) (see Figure 2) [48,49,50]. The EMG system selected was the Myo Armband (Thalmic Labs) that recorded eight differential channels at a sample rate of 200 Hz. The signals acquired were sent via Bluetooth low energy (BLE) to the selected embedded platform, a Jetson Nano development kit, for the digital signal processing stage. The access to data followed the Generic ATTribute Profile (GATT) protocol, where the Myo system is the server that is subscribed to the GATT client.

The bracelet was located in the forearm 2 cm below the upper edge of the cubital fossa. By keeping a constant location of the sensor and the user’s skin clean, the measures maintained high repeatability. Five healthy volunteers (three males and two females) between 20 to 29 years of age, who did not present any alteration in hand motor function, performed the movements of interest. The subjects were all right-hand dominant, with an average forearm circumference of 26.0 cm ± 3.5 cm. They were asked to perform 10 repetitions of each movement in five different series (10 repetitions × 5 movements × 5 series). Each repetition lasted 2 s, and a resting period of 2 min was awarded between the series to prevent fatigue. A screen connected to the Jetson Nano showed the sequence of instructions to guide the volunteers during the experiment. A total of 1250 observations were obtained, 250 per gesture.

The recorded EMG signals did not show power line noise; thus, the broadband signal (0–100 Hz) was used. The segmentation of hand movements was defined by the experiment with nonoverlapped windows of 2 s.

### 2.2. Feature Extraction

According to the literature and our previous work [6], we selected features of the EMG signals in the time domain for gesture identification purposes. The five features for each of the eight channels were mean absolute values (MAV), Willison amplitude (WAMP), variance (VAR), wavelength (WL), and zero-crossing (ZC) (Table 1). For each movement, a 1 × 40 feature vector was obtained.

Because the difference in order of magnitude of features varied from $10^{2}$ to $10^{5}$, we normalized them. The method used for normalization was min–max feature scaling which rescales the individual features in the range [0,1] (see Equation (1)).

(1) $$x_{norm}=\frac{x-x_{min}}{x_{max}-x_{min}}$$

### 2.3. Gesture Classification

Machine learning algorithms were designed to classify the five hand gestures. Due to the hardware limitations of the Jetson Nano processor, we selected classic machine learning methods: (i) support vector machine (SVM), (ii) K-nearest neighbors (KNN), and (iii) linear discriminant analysis (LDA). From the 1250 observations, 70% were used for training and validation of the algorithms, and the remaining 30% were used for evaluation of the classifiers.

A cross-validation was applied with five folds of balanced datasets for training and validation. In this process, parameters of the classifiers were optimized to improve performance: the solver for LDA; the regularization parameter, the kernel function, the number of degrees for a polynomial kernel, and the kernel coefficient for SVM; and the number of neighbors, the distance, and the type of internal algorithm for KNN.

**Figure 3:**
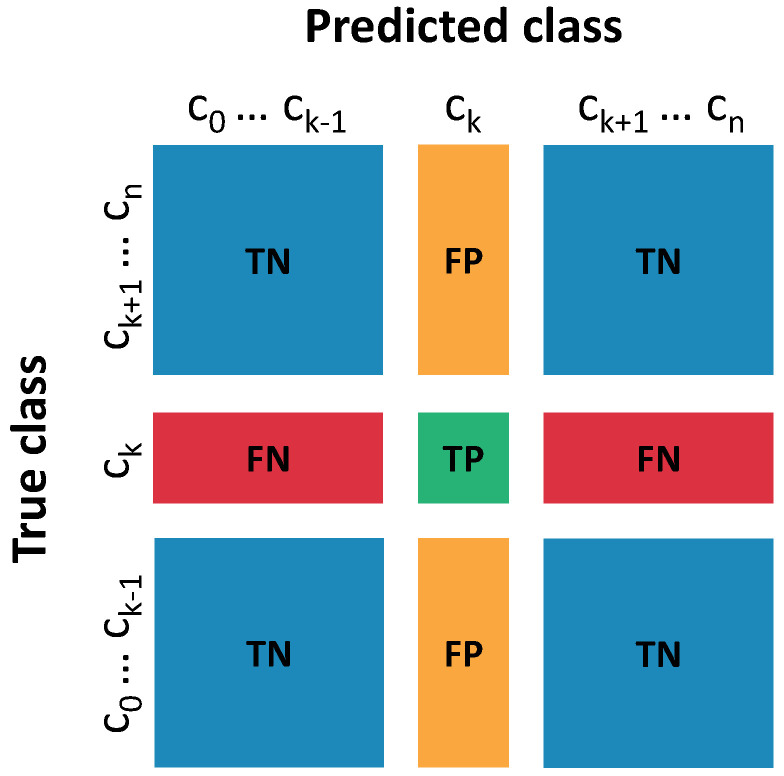
Multiclass confusion matrix. TP are true positives for class *k*. TN are true negatives for class *k*. FP are false positives for class *k*. FN are false negatives for class *k*.

Once the gesture classification models were optimized, the performance was evaluated using metrics such as accuracy, specificity, and sensitivity, given for the following expressions:

- Sensitivity: $\frac{TP}{TP+FN}$
- Specificity: $\frac{TN}{TN+FP}$
- Accuracy: $\frac{TP+TN}{TP+FP+TN+FN}$

where TP, FN, FP, and TN are defined according to the confusion matrix in Figure 3. The gesture classification stage was used to control the movement of the corresponding finger in the exoskeleton.

One of the most important contributions of our work relies on the efforts made towards the implementation of the classification process on an embedded device. Having an embedded device as the core to support these techniques allows the implementation of a flexible, light, portable, and low-power demanding device that eases the rehabilitation process, also being closer to what is desired in a final product. Such an embedded device would improve the usability for the operators of the system, also reducing the intrinsic load on the recovering patient.

We selected Google Colaboratory (or Colab) to train the models in an offline fashion. Utilizing Python, on Google Colab, we integrated different libraries to ease the training and testing processes: Scikit-learn (https://scikit-learn.org/stable/, accessed on 15 November 2022) (machine learning models), Numpy (https://numpy.org, accessed on 15 November 2022) (mathematical functions), Pandas (https://pandas.pydata.org, accessed on 15 November 2022) (data analysis), Seaborn (https://seaborn.pydata.org, accessed on 15 November 2022) (statistical data visualization), and JobLib (https://joblib.readthedocs.io/en/stable/, accessed on 15 November 2022) (model export). Although it was not particularly necessary, we were also able to train the models in our embedded platform, but with an obvious increase in the processing time.

The online classification process for all models (LDA, SVM and KNN) was implemented on the selected embedded device, the Jetson Nano development kit. In this deployment, we also utilized Python as our base language and integrated JobLib (model import) and Numpy (mathematical functions). All reported values were generated and extracted on this embedded device and later compared to the results achieved with Google Colab, as a validation step.

### 2.4. Exoskeleton

**Figure 4:**
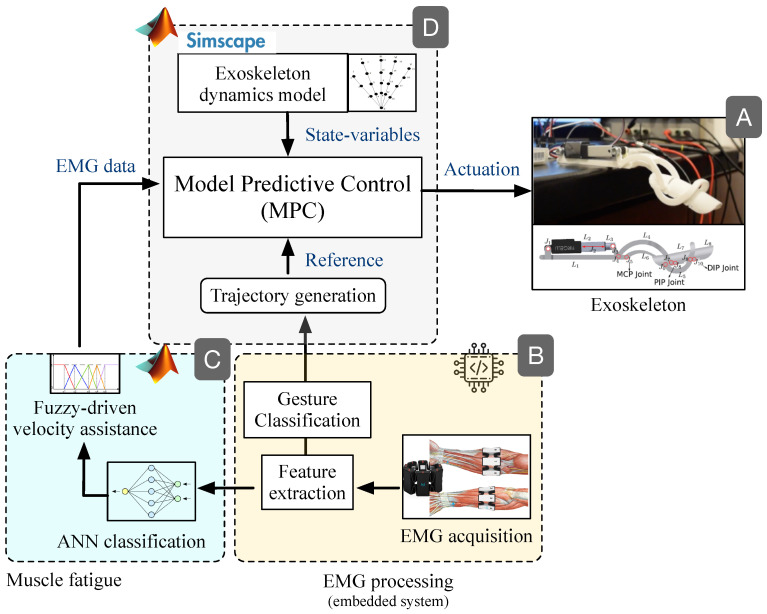
MPC closed-loop control setup. (**A**) exoskeleton prototype, (**B**) sEMG real-time gesture classification, (**C**) sEMG-based muscle fatigue characterization to define the reference velocity, (**D**) Model-Predictive-Control closed-loop controller.

The exoskeleton was developed based on the mechanical design introduced by Cui et al. [52]. Each finger of the exoskeleton had a single active degree of freedom (DOF) and three mechanical connections (links) corresponding to the three phalanges: metacarpophalangeal (MCP), proximal interphalangeal (PIP), and distal interphalangeal (DIP). Overall, the mechanism had an 8-bar with 10 joints. The thumb was not included in this study, as it was considered as a passive support for the pinch movements. Each finger was driven by an Actuonix P-series L12 linear servo-actuator with a speed range between 2 mm/s and 7 mm/s. The actuator is driven by a standard PWM-driven linear position control. The inset in Figure 4 (module A) details the proposed exoskeleton prototype.

In previous work reported in [6], we applied the well-known Newton–Euler formalism to model the exoskeleton dynamics, considering each finger as a serial chain of connected rigid body links. Equations of motion were derived by propagating joint velocities and acceleration from the base to each distal phalange. By solving the inverse dynamics problem, spatial forces and applied torques were found for each joint of the exoskeleton. The dynamics model described in [6] was validated using a ©VICON-driven motion-captured visual tracking system, where the resultant exoskeleton’s joint motion was compared against the motion generated by each individual finger while following the proposed rehabilitation trajectories. Overall, we obtained a mean squared error (MSE) of 0.33%.

### 2.5. Muscle Fatigue

In previous work [44,53], we developed a method to characterize muscle fatigue from several volunteers. The approach was aimed at assessing hand motion deficiency based on the extraction of relevant features from the EMG signals associated with the muscle condition as an indirect measure of fatigue. Muscle fatigue is defined as the process of declining output force during sustained activity. Likewise, fatigue can be detected by extracting EMG features that correlate higher to muscle condition changes. In addition, it can be classified into three levels: nonfatigue, transition to fatigue, and fatigue.

**Figure 5:**
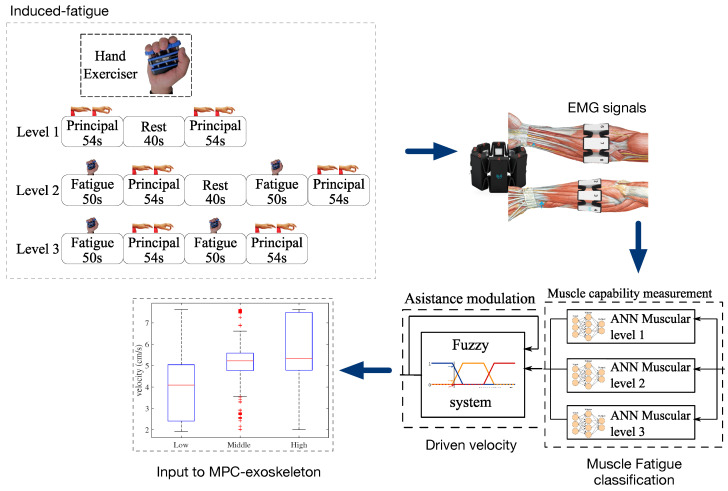
EMG-based muscle fatigue characterization.

Here, we focus on the identification of EMG signals from the forearm aimed at detecting different levels of fatigue when the hand is performing a motion. We implemented artificial neural networks (ANNs) to classify three muscular condition levels corresponding to the levels obtained in the dataset. All the subjects in this dataset do not present any hand movement disorder or impairment. Several methods for feature extraction and ranking methods from sEMG signals are implemented and evaluated in the subject groups. In particular, eleven features in time and four features in frequency-domain are extracted, followed by two different ranking methods to assess the relevance of various features for discriminating the muscular condition levels. Finally, three feedforward backpropagation ANNs were trained, one for each muscular condition level. Figure 5 presents the overall architecture for muscle fatigue characterization.

In order to achieve accurate classification of the levels, signal filtering is required to remove external noise. The Myo Armband comes with high-pass and notch filters that enable sufficient noise filtering in the EMG signals, mostly during muscular activity. However, we encountered a strong influence on the electrical activity of the muscles during the resting stage. This influence was reduced by applying the z-score standardization method. After the proper filtering, the EMG signals were segmented into windows of 1 s with an overlapping of 30%.

We defined 9 characteristics to form the feature vector; 5 features in the time domain and 4 in the frequency domain. In this sense, the feature vector ψ has 100 elements formed by the 9 extracted features for the 8 channels of the Myo device plus other 28 features for muscle coactivation. Table 2 presents the mathematical formula for the extracted features.

### 2.6. Model Predictive Control (MPC)

The model predictive control (MPC) technique allows establishment of the driven signal for the exoskeleton and adaptation of the system based on the EMG signals. Furthermore, any change in the muscular condition can be predicted, by following an iterative finite-horizon optimization of the system, considering both the dynamics equations of motion of the exoskeleton and the input EMG data [54]. In this regard, Table 3 summarizes the most relevant design parameters of the MPC controller [51,55].

Since the optimization model is highly dependent on the state variables of the exoskeleton, we used the Matlab Simscape Multibody Dynamic Toolbox *©* to compute the corresponding equations of motion in order to feedback the several state variables such as the joint positions, velocities, and moments. The MPC optimization algorithm uses the feedback to minimize the mean square error (MSE) between the actuation controller output and the reference signal. Figure 4 details the proposed closed-loop control scheme.

The MPC is primarily driven by a velocity reference, which is defined by applying cubic-spline interpolation methods based on the EMG-based gesture classification shown in module B from Figure 4. This reference is the therapy motion that drives the exoskeleton. Further details regarding EMG data interpolation and trajectory generation can be found in previous work reported in [44].

In addition to the reference velocity, the MPC also requires the exoskeleton’s state variables, concretely, the positions and velocities controlled by the linear actuator. Both variables are used for the MPC constraints shown in Table 3, but only the velocity was used as the manipulated variable. The MPC was trained by using a velocity reference ramp signal with a slope of 0.1 (speed increase given by the fuzzy model), within a range between 0 mm/s and 7 mm/s. This enables the MPC to learn the kinematics range of motion and the rate of change between velocities. In addition, a predominant tracking in the speed over the position was achieved, being key to the goal of actively assisting the patient.

Furthermore, the MPC configuration allows a direct coupling with EMG data associated with different velocity levels to counteract muscle fatigue, as shown in module C from Figure 4. In previous work reported in [44,56], we used a feedforward back propagation artificial neural network (ANN) to classify the EMG extracted features into three levels of muscular condition change, correlated with muscle fatigue levels, e.g., low, normal, and high fatigue. These levels were input into a Fuzzyfication model composed of several velocity-defined membership functions translated to 1647 fuzzy rules that generate the output velocity. Depending on the combination of the ANN inputs, the logic system increases, decreases, or maintains the output value. When the ANN inputs indicate that the muscular effort is high, the model will increase the output velocity while the ANN inputs do not change. Otherwise, the output velocity decreases when the ANN inputs indicate minimal muscular effort. Overall, a Sugeno-Fuzzy inference MISO model is in charge of these logical combinations based on the EMG classification generated by the ANN. As mentioned, further details are found in [44,56].

Here, the EMG-based velocity data are used as another input to the MPC controller, with the aim of predicting muscle fatigue to modulate the actuation signal and compensate the assistance motion accordingly. As observed in module D from Figure 4, the actuation output is generated as a function of motion reference, the EMG-based velocity assistance, and the state variables of the system. Using the MPC constraints parameters, the EMG-derived velocity input has a priority in terms of tracking, allowing the controller to properly compensate muscle fatigue independently of the therapy motion reference.

## 3. Results

### 3.1. Gesture Classification

The EMG signal characteristics presented in Section 2.2 were extracted and used as the input of the classifiers. The feature vector of size 1×40 was defined as

$$\left[MAV,WAMP,VAR,WL,ZC\right]$$

where each feature denoted a vector that contains the corresponding characteristic for each of the eight channels.

Three different classifiers were implemented (LDA, SVM, and KNN), where 70% of the events, corresponding to 175 examples per gesture (balanced events), were used for training and validating the model parameters.

**Figure 6:**
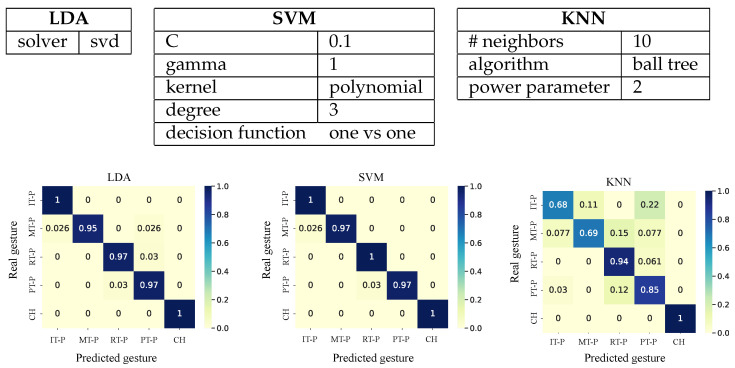
Confusion matrices of the three trained models applied to the subject. Gesture labels are defined in Figure 2.

In training, the three classifiers obtained a correct classification rate (accuracy) greater than 0.95, 0.97, and 0.78 for LDA, SVM, and KNN, respectively. The SVM classifier performed best with 0.98±0.02 accuracy, followed by LDA with 0.97±0.02 and KNN with 0.89±0.11. The gestures that obtained the highest and lowest correct prediction rate on average were the closed hand and the ring-thumb pinch with 1.00±0.00 and 0.88±0.12, respectively. Figure 6 shows the results.

Accuracy with the highest average index was the closed hand with 0.97±0.10, while the gesture with the lowest average correct index was the heart-thumb pinch with 0.72±0.15. In the case of the average rate of the classifiers, the LDA was positioned with the best performance of 0.93±0.03, followed by the SVM with a performance with 0.86±0.03. Ultimately, KNN returned 0.69±0.04.

**Figure 7:**
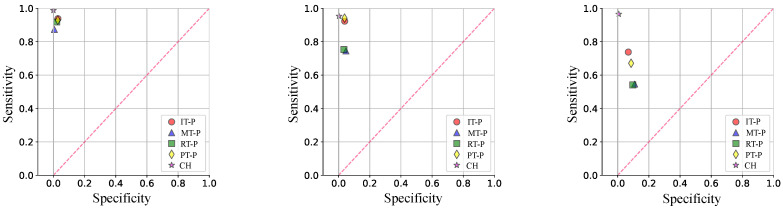
ROC data obtained from the classification of hand movements. The results presented are divided into the three implemented classifiers: LDA, SVM, and KNN. Gesture labels are defined in Figure 2.

Figure 7 shows the spread between specificity and sensitivity, similar to what is obtained from an ROC curve (receiver operating characteristic) obtained in the evaluation phase for each model and gesture. As shown in the figure, the heart-thumb and ring-thumb pinch exercises (blue triangle and green square) did not have the highest sensitivity values, with mean values of 0.929, 0.863, and 0.696 for LDA, SVM, and KNN, respectively. In the case of specificity, the gestures have an average value very close to each other in each classifier. It is highlighted that the closed-hand gesture presents a high specificity. Regarding each classification model, the LDA obtained the highest sensitivity and the highest specificity. On the other hand, KNN presents the lowest sensitivity and specificity. Table 4 shows the metrics mentioned for each subject with respect to the classifier with the best performance, that is, the LDA.

### 3.2. MPC Control of the Exoskeleton

**Figure 8:**
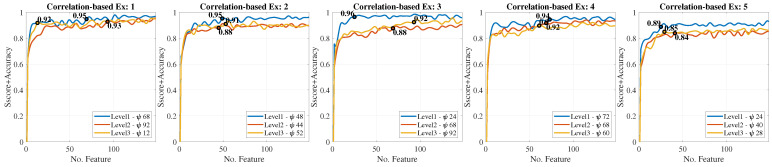
Receiver operating characteristic (ROC) curves obtained for EMG-based muscle fatigue classification using ANNs, according to the setup presented in Figure 5. Results were obtained for each motion exercise (Ex) based on the gestures from Figure 2.

Experiments were conducted to analyze the performance of the ANNs and the Fuzzy system used to classify muscle fatigue according to the three levels defined in Section 2.5. The ANN-based classifiers were trained with all features and the number of hidden layers (HL) were modified. Therefore, we tested three ANNs per level. The statistical validation metric used to compare and analyze the performance of the classifiers was the sensitivity, specificity, and the normalized mean square error (NMSE). Figure 8 shows ROC curves for each muscle condition level. We observed that both false predicted rate and true predicted rate increased for all the levels and ANN classifiers.

For the level 1, the classifier with two hidden layers obtained better specificity and sensitivity values. For the level 2, better results were obtained with three hidden layers. For the level 3, there is not a significant differences among the tested ANNs. Overall, the level 1 obtained a better relation between the true predicted rate and false predicted rate.

Based on the reported numerical results, the ANN-based classifier with one hidden layer was selected as the input for the Sugeno Fuzzy Inference System (SFIS). This selection was mainly based on the lowest NMSE and fast computational performance.

**Figure 9:**
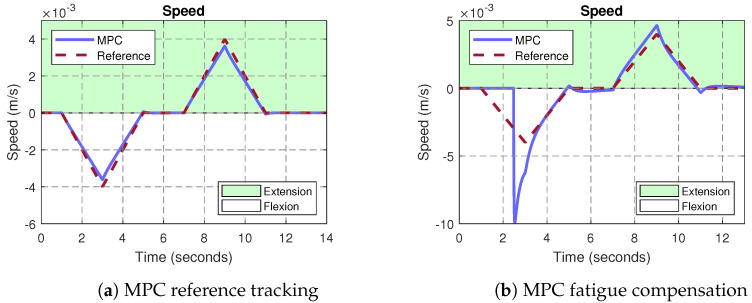
MPC−driven exoskeleton velocity control. The input reference was defined with accelerated sections, considering both positive and negative slopes. In (**a**), the MPC tracks the desired velocity profile for both finger flexion and extension therapy motions. In (**b**), the MPC considers an EMG-based velocity input associated with muscle fatigue (∀t:0⩽t⩽5), forcing the MPC controller to counteract the assistance motion by given priority to the EMG input data rather than the reference tracking.

As detailed in Figure 4, the output velocity generated by the fuzzy model is used as an input to the MPC controller. This EMG-based-driven velocity is then used by the controller to counteract muscle fatigue issues. Therefore, the performance of the MPC controller was evaluated in terms of the reference tracking error, and the muscle fatigue compensation. The mean squared error (MSE) metric was used to that purpose. In Figure 9a, a ramp-based velocity signal for flexion and extension was used as a reference, which translated into a quadratic (accelerated) position. This test was carried out in order to observe the behavior of the MPC under constant acceleration. The predominant reference to the MPC corresponds to the velocity reference trajectory rather than the EMG-based input generated by the module C in Figure 4. In terms of tracking, the results reported a maximum MSE of $4.96\times 10^{-8}$. Table 5 shows the MSE numerical results for different profiles of input velocities, corresponding to seven repetitions of opening and closing finger motions. As observed, the MPC obtained insignificant tracking errors, even for input references up to 7 mm/s. It is important to highlight that the Actuonix P-series L12 linear servo-actuator comes with an integrated low-level position control loop. In this regard, the proposed MPC controller is a high-level loop in charge of regulating the actuator’s velocity, by generating ramp-based references to the integrated low-level position control. As a result, our system is able to track the desired input velocity reference, but also to control the actuator’s linear position. In this sense, the small errors observed in Figure 9a during the changes in acceleration at t=3 s and t=9 s, respectively, are due to the integrated low-level position control, since the actuator detects minimum changes of 0.3 mm.

In Figure 9b, the same input reference was applied; however, the predominant tracking was assigned to the EMG-based input rather than the velocity reference trajectory generated by the module B in Figure 4. Under this configuration, the proposed MPC controller was integrated with the fuzzy-driven module C described in [44]. As observed, ∀t:0⩽t⩽5, the MPC discards the velocity reference, forcing the exoskeleton to compensate the assistance motion based on the EMG input associated with muscle fatigue. In this case, the controller increases the driven-velocity of the therapy up to 10 mm/s during the flexion motion in order to counteract a possible compromise of the patient’s muscle.

**Figure 10:**
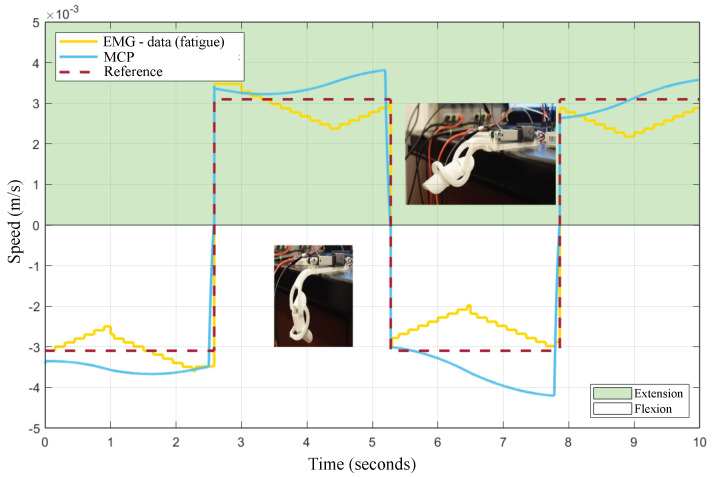
MPC−driven muscle fatigue compensation based on EMG input data.

Further experiments were conducted to analyze the EMG-based muscle fatigue compensation provided by the MPC. Figure 10 shows the results. In this test, the velocity reference trajectory was defined as PWM (pulse width modulation) instead of the ramp signal used in the previous experiment. The yellow-colored line corresponds to the velocity assistance calculated from the EMG-data, after the classification of the three levels of muscular fatigue, as explained in module C from Figure 4. As mentioned, the MPC determines an output driven-velocity between the trajectory reference (red line) and the EMG input reference (yellow line), given the tracking priority to the EMG-based muscle fatigue input. This functionality is reflected by the blue line, which counteracts the behavior exhibited by the EMG data (opposite motion direction), when the velocity of the fuzzy system is less than the reference. Contrarily, when the fuzzy-driven assistance velocity is higher than the reference, both responses exhibit a direct relationship.

Furthermore, the MPC was also able to predict the changes from flexion–extension–flexion, as observed at t=2.5 s, t=5.2 s, and t=7.8 s, respectively, improving the motion assistance provided by the exoskeleton.

Finally, the numerical results are consigned in Table 6 by comparing both the simulation and experimental tests with the exoskeleton’s testbed. As detailed in both scenarios, the correlation metrics obtained during reference tracking (nonfatigue) and those with the EMG-based fatigue input demonstrate the performance and accuracy of the proposed MPC control system for smart rehabilitation assistance.

## 4. Discussion

Three different classifiers (LDA, SVM, and KNN) were implemented to classify hand movements based on electromyography (EMG) features. The proposed classifiers were evaluated based on the accuracy, sensitivity, and specificity. During training, the SVM classifier obtained the highest accuracy (0.98), while LDA obtained the highest average performance rate (0.93). The closed-hand gesture obtained the highest correct prediction rate (1.0), while the heart-thumb pinch obtained the lowest. The LDA classifier had the highest sensitivity and specificity and performed the best overall. The LDA results in terms of sensitivity, specificity, and accuracy (above 0.9) demonstrate the effectiveness of EMG-based gesture recognition for prosthetic devices and rehabilitation purposes.

For muscle fatigue, we followed comprehensive methods and protocols from the specialized literature in this field [57,58]. Since we are not still working on real post-stroke patients, we relied on 18 healthy volunteers to emulate possible scenarios of muscle fatigue, according to the three levels of muscular condition defined in Figure 5. In order to induce muscle fatigue in our volunteers, we used one gesture: pinch-thumb grip, as shown by the insets in Figure 5. As mentioned, this EMG-based fatigue characterization was conducted on previous work reported in [53]. For instance, our approach aims to assess the subject’s muscular capability associated with the muscle condition, as an indirect measure of fatigue, considering that muscle fatigue is defined as the process of declining output force during sustained activity [57]. It is worth highlighting that most of the existing body of work classified the fatigue for the upper and lower limbs in particular [58,59,60], while few works classified muscular fatigue for the hand [61,62]. In Figure 8, ROC curves were obtained to demonstrate the accuracy of detecting EMG-driven muscle condition changes, according to the levels of muscle fatigue previously defined, by combining the hand exercises and EMG acquisition protocols from Figure 2 and Figure 5. In this paper, we took a step further, using both EMG datasets as inputs to the model predictive control (MPC), in order to trigger the exoskeleton’s assistance based on increasing or decreasing the velocity of the exercise motions.

The proposed MPC approach enabled the exoskeleton to adapt the driven rehabilitation velocity, according to the muscle effort detected from the EMG dataset, achieving correlations of >0.9 with the EMG-based velocity input. More importantly, the proposed system was also able to predict a change in the muscular condition to properly compensate through the exoskeleton. In terms of reference tracking, the MPC obtained negligible mean square errors. Although the simulation models and the experimental tests did not consider external factors such as the payload caused by the patient’s hand, the results reported in this paper are an important step towards the precise and optimal assistance under real clinical scenarios.

The performance of our proposed MPC controller was evaluated in terms of the reference tracking error and the muscle fatigue compensation using the EMG-based-driven velocity. The MPC obtained insignificant tracking errors, even large input references (up to 7 mm/s for ramp reference). The MPC was also able to predict changes with minimum error from flexion–extension–flexion (speed polarity change on PWM reference), improving the motion assistance provided by the exoskeleton. The proposed MPC control system for smart rehabilitation assistance demonstrated accurate and efficient performance for both nonfatigue and EMG-based fatigue inputs.

Most of the body of work in this field reports the use of adaptive controllers driven solely by the EMG signals. Here, our control technique allows for two input references: the joint trajectory of the rehabilitation exercise and the estimation of velocity according to the detected muscular effort. These multiple inputs not only allowed for an outstanding precision in the tracking, but also enabled the MPC to seek a balance between the desired rehabilitation and the EMG-based muscular effort, as observed in the results reported by Figure 10.

## 5. Conclusions

Our study validates the performance of a system for real-time identification of hand gestures from EMG signals implemented in an embedded system, as well as a closed-loop control system developed in an exoskeleton for compensating the effects of muscle fatigue in hand joint rehabilitation therapies.

The proposed MPC approach enables the exoskeleton to modulate the driven rehabilitation velocity according to the muscle effort detected from the EMG dataset, and the system also predicts a change in the muscular condition to properly compensate through the exoskeleton. To the best of the authors’ knowledge, applying a model predictive control (MPC) approach with two input references is a novel work in the field, enabling the exoskeleton to accurately track and apply the therapy reference while considering muscular fatigue disturbances. As a result, the controller triggers the assistance when needed, while softly forcing the patient to fulfill the therapy exercise.

Upcoming work will be focused on implementing the fuzzy model and the embedded MPC into a system on chip (SoC) platform, since neural engine cores will increase the computational response of the MPC controller while enabling machine learning models to process EMG signals in real time.

## Figures and Tables

**Table 1:** Time features extracted from the EMG signals.

Feature	Formulation
Mean Absolute Value (MAV)	$\frac{1}{N}\sum_{i=1}^{N}\left|x_{i}\right|$
Willison amplitude (WAMP)	$\begin{array}{c} \sum_{i=1}^{N}f\left(\left|x_{i}-x_{i+1}\right|\right)\\ f\left(x\right)=\left\{\begin{array}{cc} 1 & x\geq 5\mathrm{mV}\\ 0 & x<5\mathrm{mV} \end{array}\right. \end{array}$
Variance (VAR)	$\frac{1}{N-1}\sum_{i=1}^{N}x_{i}^{2}$
Waveform length (WL)	$\sum_{i=1}^{N-1}\left|x_{i+1}-x_{i}\right|$
Zero-crossing (ZC)	$\sum_{i=1}^{N-1}f\left(x_{i+1},x_{i}\right)$ $f\left(x_{i+1},x_{i}\right)=\left\{\begin{array}{cc} 1, & \mathrm{if}\;x_{i+1}>0\;\mathrm{and}\;x_{i}<0\\ 1, & \mathrm{if}\;x_{i+1}<0\;\mathrm{and}\;x_{i}>0\\ 0, & \mathrm{otherwise} \end{array}\right.$

**Table 2:** The features extracted from each sEMG signal. $x_{i}$ represents the EMG signal in a segment *i*, *N* denotes length of the EMG signal. Assuming that the frequency spectrum is divided into *M* frequencies, $f_{j}$ represents the *j*th frequency of the signal’s spectrum 1≤j≤M, and $P_{j}$ the power of $f_{j}$.

Acronym	Name	Domain	# Feat.	Equation
ACNI	Cumulative integration	time	1	$\frac{1}{N}\sum_{i=1}^{N}\frac{x_{i-1}+x_{i}}{2}△i$
ZC	Zero crossing	time	1	$$\begin{array}{c} \sum_{i=1}^{N-1}sgn\left(x_{i}x_{i-1}\right)\\ \cap |x_{i}-x_{i+1}|\geq \mathrm{threshold}\\ sgn\left(x\right)=\left\{\begin{array}{cc} 1, & \mathrm{if}x\geq \mathrm{threshold}\\ 0, & \mathrm{otherwise} \end{array}\right. \end{array}$$
MTW	Multiple time window	time	6	$$\begin{array}{c} \sum_{i=0}^{N-1}{\left(W_{i}x_{i}\right)}^{2}\mathrm{Hamming}\\ \sum_{i=0}^{N-1}W_{i}x_{i}^{2}\mathrm{Trapezoidal}\\ \sum_{i=0}^{N-1}W_{i}x_{i}^{2}\mathrm{Slepian} \end{array}$$
CFM	Contraction force muscular	time	1	$\frac{V_{RMS}}{V_{RMS(rest)}}$
MCA	Muscle co-activation	time	28	$\frac{V_{RMS(m)}}{V_{RMS(j)}}$
ASD	Average of spectral density	frequency	1	$\frac{1}{N}\sum_{j=1}^{M}P_{j}$
MNF	Mean frequency	frequency	1	$\frac{\sum_{j=1}^{M}f_{j}P_{j}}{\sum_{j=1}^{M}P_{j}}$
MDF	Median frequency	frequency	1	$\sum_{j=1}^{MDF}P_{j}=\sum_{j=MDF}^{M}P_{j}=\frac{1}{2}\sum_{i=1}^{M}P_{j}$
PR	Power rate	frequency	1	$\frac{max(P)}{min(P)}$

**Table 3:** Selection criteria of design parameters for MPC.

Parameter	Value	Additional Considerations
Sample Time (ts)	0.001 s	The plant works at that sample time.
Prediction Horizon (HP)	20	
Control Horizon (HC)	2	The higher its value, better response but greater the computational load.
Constraints (C-MV and C-MO)	C-MV = −inf,inf C-MO = −0.007, 0.007 ms${}^{-1}$	Soft constraints: can leave the range minimally Hard constraints: cannot leave the range Recommended: not all constraints hard, optimal mathematical expression could not be found.
Weights (W-MV and W-MO)	W-MV = 0 and W-MO = 0.135	Controls the deviation of the manipulated variable from the reference.
State-Estimator (SE)	Faster or slower	Faster: faster response and shorter settling time, but higher computational load.
Close-loop performance (CLP)	Robust or aggressive	Robust: less peak and allows for smoother towards the reference. Aggressive: Movement more abrupt.

**Table 4:** Sensitivity, specificity, and accuracy values during training and evaluation of the LDA model for each volunteer.

	Training			Test		
	Sen.	Spe.	Acc.	Sen.	Spe.	Acc.
Volunteer 1	0.98	0.99	0.98	0.96	0.99	0.96
Volunteer 2	0.97	0.99	0.97	0.93	0.98	0.94
Volunteer 3	0.97	0.99	0.97	0.89	0.97	0.90
Volunteer 4	0.97	0.99	0.97	0.94	0.98	0.94
Volunteer 5	0.95	0.97	0.94	0.90	0.97	0.90
Average	0.97	0.99	0.97	0.92	0.98	0.93

**Table 5:** Therapy of flexion and extension of the finger with variable speed and accelerated positions (7 repetitions). MSE for MPC controllers of position and speed for 7 opening/closing cycles, varying $v_{in}$.

	Position	Speed
2 mm/s	1.7161 × 10${}^{-7}$	3.5368 × 10${}^{-8}$
3 mm/s	1.7062 × 10${}^{-7}$	4.1654 × 10${}^{-8}$
4 mm/s	1.7617 × 10${}^{-7}$	4.5063 × 10${}^{-8}$
5 mm/s	1.8025 × 10${}^{-7}$	4.7184 × 10${}^{-8}$
6 mm/s	1.8367 × 10${}^{-7}$	4.8616 × 10${}^{-8}$
7 mm/s	1.8631 × 10${}^{-7}$	4.9636 × 10${}^{-8}$

**Table 6:** Expected muscle fatigue compensation performance measures between MPC and Ref-Fuzzy (EMG).

	Simulation Results		Experimental Results	
	Non-Muscle Fatigue	Muscle Fatigue	Non-Muscle Fatigue	Muscle Fatigue
Correlation	Flexion = 0.95 Extension = 0.84	Flexion = 0.96 Extension = 0.99	Flexion = 0.95 Extension = 0.96	Flexion = 0.91 Extension = 0.95
MSE	8.94 × 10${}^{-7}$	2.57 × 10${}^{-6}$	9.48 × 10${}^{-7}$	1.01 × 10${}^{-6}$
RMSE	9.45 × 10${}^{-4}$	1.60 × 10${}^{-3}$	9.74 × 10${}^{-4}$	1.00 × 10${}^{-3}$

## Data Availability

Data are unavailable due to privacy or ethical restrictions.
